# COVID and the Renin-Angiotensin System: Are Hypertension or Its Treatments Deleterious?

**DOI:** 10.3389/fcvm.2020.00071

**Published:** 2020-04-23

**Authors:** Florian Zores, Mathieu E. Rebeaud

**Affiliations:** ^1^Groupe Médical Spécialisé, Strasbourg, France; ^2^DBMV, Faculty of Biology and Medicine, University of Lausanne, Lausanne, Switzerland

**Keywords:** COVID-19, SARS-CoV-2, ACE2, angiotensin, renin, 2019-nCoV

## Introduction

Since its outbreak in December 2019, Severe Acute Respiratory Syndrome CoronaVirus 2 (SARS-CoV-2) has spread worldwide and is considered a pandemic. Coronavirus disease (COVID-19) can lead to acute respiratory distress syndrome (ARDS) or death. Many efforts have been made to identify risk factors predisposing to a severe issue. In the first SARS-CoV epidemic in 2002, hypertension was noted in 9/19 patients who died from SARS-CoV in Toronto (1). In the two largest cohorts of SARS-CoV-2 published, hypertension is the most common comorbidity in patients with severe disease or in those who died or were ventilated (2, 3). Nevertheless, these data are not adjusted for age, although age appears to be a strong predictor of adverse outcome (4) and hypertension is a very common finding in older patients. Finally, cohort studies only show correlation, not causality. In this paper, we hypothesize that the reductions in Angiotensin-Converting Enzyme 2 (ACE-2) observed in hypertension and obesity can explain many abnormalities observed in SARS-CoV-2 and question the role of treatments interfering with ACE2.

## ACE2 in the Cardiovascular System

Like SARS-CoV, SARS-CoV-2 fuses with human cells after the receptor-binding domain of its S (Spike) protein binds with Angiotensin-Converting Enzyme 2 (ACE-2), an enzyme located on membrane of lung alveolar epithelial cells, renal tubular epithelial cells, enterocytes of the small intestine, and arterial and venous endothelial cells of the kidney (5–10). Cardiomyocytes, fibroblasts, endothelial cells, and pericytes account for the vast majority of cells expressing ACE2 in the heart (10).

**Figure 1:**
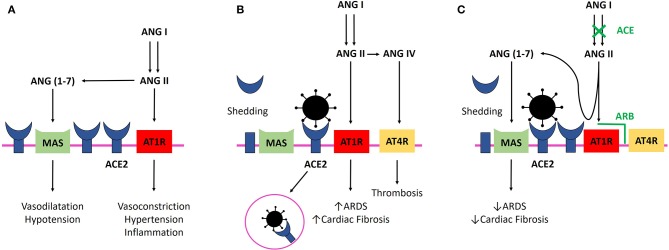
**(A)** ANGII binding to AT1R elevates blood pressure and promotes inflammation. ACE2 inactivates ANGII by converting it to ang-(1–7) and negatively regulates the renin-angiotensin system, promoting vasodilatation and hypotension. **(B)** SARS-CoV-2 infection. Binding of SARS-CoV-2 with ACE2 leads to their internalization and to ACE2 shedding by ADAM17 (enzyme not shown). Lower availability of ACE2 results in a lower rate of ANGII degradation and excessive stimulation of AT1R, which facilitates ARDS and myocardial injury. Binding of ANGII to AT1R leads to membranous ACE2 internalization, decreasing ACE2 availability even more (not shown). Excessive ANGII is metabolized to ANGIV, which binds to AT4R and promotes thrombosis. Virus replication could also reduce cellular ACE2 expression (not shown). **(C)** SARS-CoV-2 infection and ACEi/ARB treatment. ACEi and ARB upregulate ACE2, and freer ACE2 remains after viral binding. ANGII is still degraded by ACE2 in its beneficial metabolite Ang-(1–7), and AT1R and AT4R are less stimulated. ANGII binding on AT1R prevention with ARB and ANGII synthesis decrease with ACE lead to less AT1R stimulation and persistent interaction with ACE2, avoiding ACE2 internalization. ACE2, Angiotensin Converting Enzyme 2; ACEi, Angiotensin Converting Enzyme Inhibitor; ang-(1–7), Angiotensin-(1–7); ANGII, angiotensin II; ANGIV, angiotensin IV; ARB, Angiotensin Receptor Blocker; ARDS, Acute Respiratory Distress Syndrome; AT1R, Angiotensin II Type 1 Receptor; AT4R, Angiotensin II Type 4 Receptor; SARS-CoV-2, Severe Acute Respiratory Syndrome CoronaVirus 2.

ACE-2 is a monocarboxypeptidase homologous to Angiotensin-Converting Enzyme (ACE) whose active site is exposed at the extracellular surface (8, 11). ACE cleaves angiotensin I (ANGI) to generate angiotensin II (ANGII), which binds to and activates Angiotensin Type 1 Receptor (AT1R) to constrict blood vessels and increase salt and fluid retention, thereby elevating blood pressure. ACE2 inactivates ANGII by converting it to angiotensin-(1–7), which has a vasodilator effect when binding to Mas receptor (12) (Figure 1A). Moreover, ACE2 cleaves ANGI into angiotensin-(1–9) (albeit with lower affinity than for ANGII), which is further converted into angiotensin-(1–7) by ACE (12). Thus, ACE2 negatively regulates the renin-angiotensin system and modulates the vasoconstriction, fibrosis, and hypertrophy induced by that system (8, 11). In rats, ACE2 deficiency worsens hypertension when ANGII is in excess (8, 13). In human, gene expression and/or ACE2 activity is lower in hypertensive patients than in normotensive ones (13).

Conversely, ANGII negatively regulates ACE2. AT1R and ACE2 physically interact to form complexes on the cell membrane in the absence of excess Ang II (11). ANGII increase separates AT1R and ACE2 on the cell surface and leads to ACE2 internalization and lysosomal degradation through an AT1R-dependent mechanism (11, 13). Moreover, cellular ACE2 can be cleaved and released (shedding) by the metalloproteinase ADAM17, which is upregulated by ANGII (14). The soluble form of ACE2 circulates in small amounts in the blood, but its physiological role remains elusive, and shedding could be only a mechanism to regulate ACE2 activity on the cell surface (15).

Notably, it has been shown that infection with SARS-CoV can be blocked with soluble ACE2 molecules (6), and some have hypothesized that a soluble recombinant form can be used to overwhelm SARS-CoV-2 to prevent its binding to cellular ACE2 (16). Recombinant human ACE2 has been tested in a phase 2–3 trial in ARDS with interesting results (17), and a pilot trial has recently been launched in COVID-19 (NCT04287686).

ACE inhibitors (ACEi) and AT1R blockers (ARB) are two classes of drugs that are widely used in medicine to treat hypertension or heart failure. ACEi and ARB upregulate ACE2 expression on the cell surface, and ACE2 activity is not prevented by ACEi (8, 11, 18). Accordingly, patients treated with ACEi/ARB could have a higher level of membrane-bound ACE2, providing a more potent binding site to COVID-19 S protein. Nevertheless, in the absence of excess ANGII (either by reduction of ANGII synthesis by ACEi or by AT1R blockade thanks to ARB), AT1R is thought to interact with ACE2 (11). This interaction could reduce the affinity of COVID S protein to ACE2 and then reduce COVID-19 viral entry (11).

In the heart, ACE and ACE2 balance Ang II levels and ACE2 is known to be cardioprotective (8). ACE2 loss leads to a decrease in myocardial function in rodents, likely mediated by ANGII-induced oxidative stress and inflammation through AT1R, but it is unknown whether excess ANGII has a role in an acute setting (8, 19). This decrease is corrected by ARB or ACEi, and these drugs rapidly increase ACE2 activity and mRNA expression in the heart of rats (8, 20). Evidence for such an increase in humans is lacking, but studies checked for variation in the circulating level rather than the tissular level of ACE2 (21). In human failing heart, ACE2 expression is increased, correlating with disease severity, and is thought to be a compensatory mechanism (8, 10).

## Role of ACE2 in SARS-COV-2 Infection

SARS-CoV-2 has a 10–20-fold higher affinity for ACE2 than does the 2002 SARS-CoV (22). An increased abundance of cellular ACE2 is associated with a higher susceptibility to SARS-CoV infection in mice (23). However, in both heart and lung, binding of the SARS-CoV to ACE2 leads to the loss of ACE2 by ACE2 internalization with the virus and ACE2 shedding (7, 9, 14). Lower availability of ACE2 results in a lower rate of ANGII degradation. In rodent lungs, excess ANGII binding to AT1R increases pulmonary vascular permeability and neutrophil accumulation and enhances lung injury (7, 24) (Figure 1B). Thus, decreased ACE2 expression promotes increased lung injury and ARB prevents it by limiting ANGII binding to AT1R (7, 8, 24, 25) (Figure 1C). This hypothesis is supported *in vivo* by the increased frequency of severe ARDS in patients infected with SARS-CoV with higher levels of ACE determined by genetic predisposition, leading to higher levels of ANGII (26), and by the correlation between viral load, ANGII plasma level, and disease severity in influenza H7N5 (27) and respiratory syncytial virus infection (25). More notably, in a small cohort of patients infected with SARS-CoV-2, viral load was correlated with plasma ANGII level (28). Unfortunately, baseline treatments are unknown in this cohort, and correlation between ARDS severity and plasma ANGII level failed to reach statistical significance, maybe because of the low number of patients.

Moreover, some have suggested that viral replication by itself can reduce cellular ACE2 expression (29). This point is of importance because limitation of ANGII formation by ACEi and binding to AT1R by ARB may yet become the best ways to limit lung injuries if ACE2 is less or not synthetized following viral infection.

SARS-CoV- and SARS-CoV-2-associated cardiac injury contributes significantly to morbidity and mortality and could hit as much as a third of patients with a severe form of the disease (9, 28, 30, 31). SARS-CoV was found in the heart of a third of human autopsy hearts, with a concomitant marked reduction in cellular ACE2 (9). As in lungs, ANGII probably contributes to the deleterious effect of SARS-CoV on the heart and to SARS-associated cardiomyopathy, even if myocardial dysfunction can also be influenced by the strong immune response observed in those patients (9). Inflammatory signals are likely to suppress ACE2 transcription and down-regulate cell-surface expression of ACE2 (8). Thus, inflammatory signals could decrease the cellular susceptibility to SARS-CoV infection but increase the ANGII-mediated tissular injury. Moreover, because pericytes are supposed to play a role in myocardial microcirculation, SARS-CoV-2-induced microcirculation disorder could explain the frequent cardiac marker increase observed in hospitalized patients (2), exacerbated by the reduced oxygen supply caused by lung failure (10).

In summary, a decrease in cellular ACE2 may reduce the susceptibility of cells to SARS CoV-2 but leads to greater activation of AT1R and more severe tissue damage. In contrast, the higher the abundance of ACE2 on the cell membrane, the greater the susceptibility to viral particles but the less the damage, due to less AT1R activation occurring. This latter condition is the one provoked by ACEi/ARB treatment. On the one hand, ACE2 increase under ARB/ACEi treatment could be protective during COVID-19 because some ACE2 remains free to degrade ANGII, but on the other hand, this ACE2 increase could be deleterious by favoring cellular infection by COVID-19, leading to potent myocarditis (Figure 1C). The protective or deleterious role of ACEi/ARB in COVID-19 is harder to modelize, as ACE2 is not the only protein required for SARS-COV-2 penetration (5).

## Are ACEI and ARB Deleterious in SARS-COV-2 Infection?

It has been shown that both ACEi and ARB upregulates ACE2, and a hypothesis was proposed by several authors of a potential deleterious effect of treatment with ARB and ACEi in the course of SARS-CoV-2 infection (32, 33). Since these molecules are widely used to treat hypertension or heart failure, such a fact could be a huge matter of concern.

Obesity seems to be a major determinant of adverse outcome in COVID-19 (34). Besides the altered pulmonary function associated with obesity, it must be noted that obesity is associated with a decrease in membranous ACE2 (35, 36). Moreover, empirical observations are suggestive of an abnormally high prevalence of pulmonary embolism in patients with COVID-19 (37), and prophylactic curative anticoagulation is recommended in severe patients (38). Severe infections are a known precipitant factor for acute venous thrombo-embolism because of epithelial damage and platelet and endothelial cell dysfunction, but does it by itself explain the observed high prevalence of pulmonary embolism in these patients? When ANGII is increased, it can be metabolized to angiotensin IV (ANGIV) by aminopeptidase A and binds to Angiotensin Type 4 Receptor (AT4R) (39). Multiple datasets underline the enhancement of thrombosis development by ANGII and ANGIV (40, 41), and it can be hypothesized that a reduction in ACE2 can increase thrombotic risk.

Despite the many potential cofounders, reduction in membranous ACE2 expression could be an explanation for numerous abnormalities observed in SARS-CoV-2 infection. Thus, even if both ARB and ACEi increase the level of ACE2, more ACE2 could be better rather than worse: more ACE2 remains on the cell surface after virus binding, maintaining ANGII degradation and less stimulation of AT1R. Furthermore, treatment with ARB inhibits AT1R and limits the damage induced by its overstimulation. It is not clear whether continuation or discontinuation of ARB or ACEi is a good option in COVID-19 infection, as there is a lack of clinical data to support an increased risk of contracting a severe form of COVID-19. In addition, we do not even know whether renin angiotensin system inhibitor therapy is beneficial or harmful for virally mediated lesions, and switching to other drugs may worsen the patient's condition, especially for heart failure patients with reduced ejection fraction (42). Clinical trials are ongoing to analyze the beneficial effect of LOSARTAN in COVID-19 (NCT04311177 and NCT04312009), and a trial will start soon to analyze the consequences of discontinuation or continuation of ACEi/ARB (NCT04338009).

ACEi and ARB are not the only treatments for hypertension or heart failure, but other classes only have a limited impact on ACE2. Beta blockers suppress plasma angiotensin II levels by inhibiting prorenin processing to renin and probably do not interfere with ACE or ACE2 (43). Calcium channel blockers seem to reduce ANGII-induced downregulation of ACE2, but data are limited to those presented in one paper on the effect of nifedipine on fractionated cell extracts (44). In hypertensive rats, neither thiazides nor mineralocorticoid-receptor antagonists (MRAs) improve the spontaneous low ACE2 activity (18, 45), but MRA could decrease ACE expression (18). Conversely, MRAs increase membranous ACE2 activity in patients (46) with heart failure. If the reduction of membranous ACE2 observed in hypertension and obesity plays an important role in the pathophysiology of severe COVID-19, can it be hypothesized that non-ACEi/BRA drugs (beta-blockers, calcium channel blockers, diuretics) are more likely to increase the risk of deleterious outcomes than ACEi/BRA drugs that increase ACE2 and provide theoretical protection? Data on baseline treatments are urgently needed but are lacking to date in published cohorts.

## Conclusion

The downregulation of ACE2 induced by viral binding, resulting in increased stimulation of AT1R, may be an important element in explaining severe COVID-19. Overall, the ACEi/ARB-mediated increase in ACE2 is not obviously deleterious and may even be protective. Only a well-conducted trial will provide a valid answer to this question. To date, stopping this treatment solely on the basis of presumed considerations does not seem to be a good option.

## Author Contributions

MR and FZ wrote the manuscript, conceptualized the idea, and made the figure. All authors reviewed and approved the final version of the manuscript.

## Conflict of Interest

The authors declare that the research was conducted in the absence of any commercial or financial relationships that could be construed as a potential conflict of interest.
